# Assessment of Secondary School Students’ Game Performance Related to Tactical Contexts

**DOI:** 10.2478/hukin-2014-0076

**Published:** 2014-10-10

**Authors:** David Gutiérrez, Jennifer Fisette, Luis Miguel García-López, Onofre Contreras

**Affiliations:** 1 Faculty of Education - University of Castilla-La Mancha, Spain.; 2 Kent State University, United States (OH).; 3 Faculty of Education, University of Castilla-La Mancha, Spain.; 4 Faculty of Education, University of Castilla-La Mancha, Spain.

**Keywords:** teaching games for understanding, performance based assessment, physical education, invasion games, small sided games, constraints

## Abstract

Certain limitations remain unaddressed when utilizing the Teaching Games for Understanding approach, suggesting the need for more research on authentic assessment of skill development and tactical awareness in order to guide the design of developmentally appropriate curriculum materials. This study investigated physical education students’ (n=19; age: 13.71 ± 0.4) game performance during an invasion game, specifically the relationship between their skill execution and decision-making ability. The purpose of the study was twofold: (a) to devise and implement a ‘game context’ approach to assess the game performance components and in doing so, (b) to provide information that could be used to design suitable learning progressions within tactical teaching approaches. Students’ game performance was videotaped, and measures of skill execution and decision-making were developed from observational analyses. Decision-making was measured at two levels: a) decision making restricted to the selection of technical-tactical skills (i.e., passing, moving with the ball, getting free, marking, tackling, double teaming and interception; and b) decision-making in the adaptation to the tactical contexts of the game. Participants played a 5 vs. 5 modified eight-minute team handball game. Participants scored significantly higher in penetrating-the-defense context adaptation than in keeping-the-ball context adaptation. Participants showed a higher efficiency in decision-making than in execution in most of the technical-tactical skills; including on-the-ball over off-the-ball decision-making, and in attack compared to defensive execution. The findings also revealed significant relationships between decision-making and skill execution in shooting, tackling and passing.

## Introduction

Game centered teaching games methodology has been researched and argued for by Oslin and Mitchell (2006) under the name of Game-Centered Approaches (GCAs). GCAs, such as, the Tactical Games Model (TGM) (Griffin et al., 1997) and Play Practice (Launder, 2001) are forms of the original and most well-known model, Teaching Games for Understanding (TGfU) (Bunker and Thorpe, 1982). GCAs place the games player (e.g., student, athlete) at the center of the learning experience and emphasize decision-making, critical thinking, and problem solving, which varies from more traditional teacher-centered approaches. The TGfU approach was developed in response to traditional ‘skill drill’ pedagogical approaches. TGfU originated by physical educators from the Loughborough University; firstly by David Bunker and Rod Thorpe, who presented a six-steps curricular model as a synthesis of their ideas (Bunker and Thorpe, 1982), and later, in collaboration with Len Almond, by introducing four pedagogical principles (sampling, modification-representation, modification-exaggeration and tactical complexity) that complemented the curriculum model (Thorpe et al., 1986).

### TGFU model limitations

Since the TGfU original model was not directly based on learning theories, initially there was a lack of a sound theoretical framework from where to explore the richness of the teaching and learning processes that happens when using this approach. According to Oslin and Mitchell (2006), GCAs, theoretically, have evolved from comparative studies underpinned by knowledge based theories to naturalistic inquiries teaching and learning processes situated by theories of learning. In this sense, over the past decade, different scholars have researched and framed the tenets of TGfU to constructivist theories (Light and Fawns, 2001), a situated learning theory (Kirk and Macdonald, 1998) and more recently with constraints-led and non-linear pedagogy perspectives (Renshaw et al., 2010; Tan et al., 2012). Research within this last area has grown rapidly in the last few years, especially when focusing on the effects of manipulation on task constrains (Aguiar et al., 2012; Almeida et al., 2013).

Despite these improvements, some limitations remain unaddressed, such as the need for more research to be conducted on the four pedagogical principles in order to guide the design of developmentally appropriate curriculum materials (Butler et al., 2008). Furthermore, in a recent review of GCAs literature since 2006, Harvey and Jarrett (2013) argued that there was limited research on authentic assessment of skill development and tactical awareness. One of the consequences of these limitations is that learning progressions in sport and games are not based on research data. The focus of this article is based on a research study that aims to provide evidence by using an authentic assessment instrument in the evaluation of students’ prior knowledge by providing information for how to design data-based learning progressions.

### The need for assessment related to tactical context

Potentially the most widespread learning progressions that emphasize problem solving within sport-related games are those devised by Mitchell et al. (2003; 2013). These progressions are developed for specific games such as football, cricket, volleyball, and golf. These four games represent a sport within each of the games classification system – invasion, net/wall, striking and fielding, and target games (Mitchell et al., 2013). Each of the game categories is based on different tactical goals and problems, particularly how to score, prevent scoring, and restarting game play. The tactical goals and problems in invasion games are: a) scoring on offense: keeping possession, penetrating and attacking, and transitioning from offense to defense; b) preventing scoring on defense: defending space, defending the goal, and winning the ball; and c) starting and restarting play: beginning the game, restarting from the sideline and/or endline, and restarting from violations (Mitchell et al., 2013). These tactical problems are similar in invasion games such as soccer, basketball, and football, which allows for transfer of knowledge across these games to guide the teaching and learning process. The intention of this proposal is to sequence the content in order to make games instruction developmentally appropriate for students. In this sense, the authors identify three levels of game complexity; each level is determined by the maximum number of players that is recommended (e.g., Level I: 3 vs. 3, Level II: 4 vs. 4 and Level III: 5 vs. 5). Each level includes learning of concepts, movements and skills within the aforementioned tactical problems. Game complexity increases as students’ progress through the levels.

As the whole game can be divided into different tactical problems (Mitchell et al., 2013) or action principles (Bayer, 1992), the assessment should consider efficiency of different game performance components (e.g., skill execution and decision-making) in relation to the specific problem that the player is facing in each moment of the game.

### Previous research

Despite the given importance of tactical problems in GCAs, there are only a few studies that assess and take into account prior learning of games. Among these studies there are those conducted by Blomqvist et al. (2005) and Castejón and López (2000), which assess game performance in different tactical contexts using modified games. These studies assess students’ game performance, particularly their understanding of the tactical problems, but their competency in the game cannot be completely understood, because the game form used for the assessment was not the whole game, but an invasion game modified by exaggeration that focused on a given tactical problem. On the other hand, recent studies have assessed game performance taking into account the tactical context during the whole game. In physical education, Gutiérrez and García-López (2012a) assessed sixth graders game performance in a 4 vs. 4 invasion game. In this study, participants demonstrated the highest offensive tactical awareness in penetrating the defense contexts, and the lowest scores in attacking-the-goal contexts. Within the technical-tactical-skill performance the highest scores were obtained in on-the-ball and offensive variables. Sanchez-Mora et al. (2011) studied fourth graders’ tactical knowledge. In this study, participants were assessed through a 3 vs. 3 invasion game, interviews and video analysis. The students’ game performance was assessed specifically in passing, getting free, and off the ball movements in three different tactical contexts. Sanchez-Mora and colleagues found that participants scored significantly higher in passing and dribbling, both in decision-making and skill execution, when the action took place in a penetrating-the-defense context rather than in a keeping-the-ball context. Furthermore, this approach has been also used to evaluate gender differences in tactical behavior (Gutiérrez and García-López, 2012b) and to compare decision-making between physical education students without previous learning experiences in invasion games and expert soccer players in a cross sectional study (Gutiérrez et al., 2011).

Furthermore, a recent study in soccer by González et al. (2012) contemplated the tactical contexts as a key element in the evaluation of game performance. All of the studies mentioned above, both in physical education and youth sport soccer, have used Bayer’s classification of action principles (1992) as a list of tactical contexts for assessing game performance. Related to these studies, and with the idea of developing assessment instruments that establish a connection between the contents of training and tactical development, da Costa et al. (2011) developed the FUT-SAL (System of Tactical Assessment in Soccer). This instrument considers ten tactical principles. Although the aim of this classification of tactical principles and iFUT-SAL intend to establish a “connection between the contents of training sections and assessment of the tactical development of players” (da Costa et al., 2011), they were more detailed and created with the aim of being used specifically in soccer.

The aforementioned studies, along with the present one, have the potential to link pedagogical proposals as those described by Mitchell et al. (2003) for generic invasion games, with the characteristics of each age range. This study aimed to add valuable information to reduce the above mentioned limitations. Therefore, the purpose of the study was twofold: (a) to devise and implement a ‘game context’ approach to assess the game performance components and in doing so, (b) to show how authentic assessment could be informative for the design of suitable learning progressions within tactical teaching approaches.

## Material and Methods

### Setting and participants

The study sample included 19 elementary physical education students (11 girls, 8 boys; age: 13.71 ± 0.4). Participants were selected from students with no formal training in invasion games and without any experience in officially governed competition. Subjects were evaluated through a 5 vs. 5 invasion game (i.e., team handball). Each participant took part in an 8-minute game, which was divided into two four-minute halves. Rules were minimized and modifications were made to potentially increase the subjects’ success with skill execution. The game form was selected based on the subjects’ developmental abilities and prior experience with the goal that students would be able to reach maximum achievement in the decision-making component. Small-sided games, such as 5 vs. 5, increase students’ opportunities for game involvement, both when they possess, and when they do not possess the ball (i.e., on-the-ball skills and off-the-ball movements) (Mitchell et al., 2003). The design of the modified invasion game for novices was based on those used in similar research studies conducted in educational contexts (Blomqvist et al., 2005; Nevett et al., 2001). The following structural and rule modifications were implemented in the 5 vs. 5 team handball games: a) the objective was to score goals by throwing the ball into the goal; b) moving with the ball was possible only when bouncing the ball; c) no double-dribble rule; d) stealing the ball from an opponent and physical contact was not permitted; e) after a foul, the game was restarted from the place where the infraction took place; f) throwing the ball to score from their own half of the court was not permitted; and g) only ‘1 on 1’ defense was permitted, with the same skill level attacker-defender pairs established previously by the teachers (Gutiérrez et al., 2011). Appropriate school and parental consent had been obtained to gather data that included curricular documents and video recording of students. Ethical details of the project were revised and approved by the University of Castilla-la Mancha and Castilla-la Mancha Regional Government (Reference nº: UCLM-JCCM/0144034).

### Procedures

All games were recorded with a video camera located behind and above the court. Videos were analysed after each game with the Game Performance Evaluation Tool (GPET) (García-López et al., 2013). This instrument was designed to explicitly demonstrate students’ cognitive understanding of tactical problems within GCAs. The GPET allows observers to assess the game performance decision-making and skill execution components, with regard to the tactical context in which the player is performing. Harvey and Jarret (2013), in comparison with other instruments as GPAI or TSAP, considered that due to the multi-level coding required, the GPET expanded the complexity and possible utility for research. The GPET addresses some of the limitations mentioned previously in this article by considering the efficiency of different game performance components (e.g., skill execution and decision-making) in relation to the specific problem that the player is facing in each moment of the game, by assessing game performance at two levels. The first level involves the technical-tactical skill of the players and their opponent directly implicated in their action (e.g., passing the ball to an unmarked teammate). The second level of decision-making considers the tactical-context-adaptation, which is adjusting the response to the tactical context in which the action takes place. The tactical context is determined by the scenario composed by all performers in the game that could have any influence on the game play, as well as the area where the action takes place. Table 1 summarizes and describes the coding categories. Both first level decision-making and skill execution were evaluated in the technical-tactical skills included in the first column. These variables are presented by game roles. In order to get a clearer comparison of different game aspects, variables related to technical-tactical skills were grouped in global variables (defense, attack, on-the-ball, and off-the-ball). The second column includes variables related to the second level of decision-making, i.e. tactical-context-adaptation. Tactical-context-adaptation performance was grouped in a single variable (global-context-adaptation performance) and also analysed by the three offensive tactical contexts previously described.

Skill execution was judged as successful (1) or unsuccessful (0). Decision-making was analysed at two levels. At both levels correct decision-making was coded as (1) and incorrect decision-making was coded as (0). The first level evaluated decision-making related with the execution of a specific skill or movement (e.g., correct decision making (1) would be if the player passed the ball to another player who was free from an opponent, and an incorrect decision (0) would be moving to try and get free to a space where one opponent was standing).

The second level analyzed the offensive tactical-context-adaptation through the evaluation of players’ tactical intention with regard to the tactical-context in which the action was located. Based on Bayer’s (1992) action principles classification, the GPET includes three offensive tactical-contexts: maintaining possession of the ball, penetrating-the-defense and attacking-the-goal. The tactical-contexts were coded as 1A, 2A or 3A, respectively. These abbreviations will be used throughout the manuscript.

For coding purposes, the playing time was divided into decision-making units (Nevett et al., 2001). The ending of a decision-making unit occurred in the following conditions: a) after four seconds of action; b) when the player performed a different technical-tactical skill; or c) when the offensive tactical context changed.

### Statistical analysis

The mean and standard deviation were calculated for each variable. The Kolgomorov-Smirnov test for the assumption of normality and the Levene test for homogeneity of variance or homoscedasticity showed that the sample did not meet these assumptions for all the variables in the study. Therefore, and also due to a small sample size, the Mann-Whitney U test was conducted to analyse for differences between the two samples. The Wilcoxon’s test was conducted for the two dependent samples. Lastly, the relationships between decision-making and skill execution were examined by using the Pearson’s correlation coefficient (Vincent, 2005).

## Results

The results are presented in five sections, which include descriptive scores of game performance and intra group data analyses, specifically: (a) descriptive scores and comparison of tactical context adaptation performances; (b) offensive scores and comparison of offensive skills performance (pass, move with the ball and get free) in different tactical contexts; (c) defensive scores and comparison of performance between defensive skills presented in the two defensive roles (marking and double teaming); (d) correlation between decision making and skill execution; and (e) comparison between global variables: attack/defense; on-the-ball/off-the-ball. 156 DMUs were analyzed, in which 2269 technical tactical skills were performed by the players, 859 offensive and 1410 defensive.

### Adaptation to tactical contexts

On average, each participant took part in 31.74 (*SD* = 4.98) decision-making units where the context was coded as 2A (penetrating-the-defense); 7.31 (*SD* = 4.01) decision-making units where the context was coded as 1A (keeping-the-ball); and 1.89 (*SD* = 1.99) decision-making units where the context was coded as 3A (attacking-the-goal). Subjects achieved a global context-adaptation performance of 77.34% (*SD* = 14.09) of good decisions. The context in which participants achieved better performance was attacking-the-goal (*M* = 82.82; *SD* = 30.78), while the lowest performance was in keeping-the-ball (68.39; *SD* = 20.79). Significant differences (*p*<.05) were found between scores of keeping-the-ball and penetrating-the-defense (*M* = 79.22; *SD* = 15.38). Participants did not show tactical intention nor involvement in the game (‘watcher-players’) in a 4.52% (*SD* = 5.49) of the decision-making units.

### Offensive variables

As reported in Table 2, participants in the attacker on-the-ball variables achieved higher offensive scores. Scores were higher than 90% of efficiency in the decision-making of three skills: pass decision-making in the penetrating-the-defense context (93.38%), shooting decision-making (93.8%) and moving with the ball decision-making in the keeping-the-ball context (93.75%). With the exception of passing scores in the keeping-the-ball context, participants showed higher efficiency in the decision-making component than in skill execution. The biggest difference was found also in passing, but in the penetrating-the-defense context (decision-making: 89.24; execution: 69.63).

### Defensive variables

There was a significant difference between the values of decision-making and skill execution in the defensive variables (Table 2). The most remarkable variables, due to their high percentages, were associated with decision-making: 93.5% for decision-making in blocked shots, 92.8% for decision-making for double team and 86.9% for decision-making in interceptions. Low results were compiled in the execution components: marking by the defender-on-the-ball (25.5%), blocked shot (10.4%), tackle (29.5%) and double team by the defender of the ball (30.7%). Important differences were found for each variable in the comparison of the effectiveness of decision-making and skill execution in the same technical-tactical skill.

When comparing scores obtained by the participants in the variables presented in the two defensive roles, marking and double teaming, there were significant differences in marking, both in decision making (*p* = .004) and skill execution (*p* = .022). In decision-making, there was a better result in defender-on-the-ball, while in skill execution, it was found for defender-off-the-ball. There were no significant differences in double-teaming.

### Global variables

The global variables with best scores were decision making for actions on-the-ball (*M*= 87.84% *SD*: 6.93) and decision making in offensive actions (*M* = 86.1%; *SD*: 7.34). Global variables, which reflected the lowest scores, were execution in defensive actions (*M* =34.69%; *SD*: 16.01) and execution in off-the-ball actions (*M* = 47.41%; *SD*: 19.72). The rest of the results were: decision making in defense (*M* = 79.4%; *SD*: 11.09); execution in attack (*M* = 73.22%; *SD*: 9.17); execution on-the-ball (*M* = 49.44%; *SD*: 12.01); and decision making off-the-ball (*M* = 47.41%; *SD*: 19.4). In the on-the-ball/off-the-ball comparison there were significant differences in decision making in favour of on-the-ball actions (*p* = .002). In the case for comparison between attack and defense, there were significant differences in the execution component in favour of offensive actions (*p* = .001).

### Correlation between decision-making and execution

In this analysis, decision-making and execution potential correlations were studied, both in isolated skills and in global variables. For passing, moving with the ball and getting free, the analysis was made separately in actions made in keeping-the-ball contexts and penetrating-the-defense contexts. There were significant correlations into different technical-tactical skills. This correlation was moderate (*p*<0.05) in case of shooting and tackle; and high (*p*<0.01) for passing in keeping-the-ball contexts, passing in penetrating-the-defense contexts, global passing, getting free in keeping-the-ball contexts, getting free in penetrating-the-defense contexts, global getting free, and defender-on-the-ball scores.

## Discussion

The purpose of the study was twofold: (a) to devise and implement a ‘game context’ approach to assess the game performance components and in doing so, (b) to provide information that could be used to design suitable learning progressions within tactical teaching approaches. Data on efficiency in the adaptation to the tactical contexts demonstrated that participants possessed a medium-high tactical awareness (Mitchell et al., 1994). When comparing the results by tactical contexts, the most relevant comparison in didactic terms was between the efficiency in the adaptation to the keeping-the-ball context confronted to the penetrating-the-defense context. In this comparison, there were significant differences, as players showed a higher tactical-context-adaptation when the tactical situation implied to penetrate the defense, and had difficulties to recognize those situations in which they should keep the ball. They also showed high efficiency in the identification of situations in which they should try to get a goal (3A). Therefore, this group demonstrated enhanced game understanding when the tactical problem to be solved was directly linked to the objective. Furthermore, players showed a clear goal orientation, as in the totality of the actions in which the players did not decide to shoot in an attacking-the-goal context (coded as incorrect), they made actions of penetration.

While results related to the comparison of efficiency on 1A and 2A are congruent with that reported by Gutiérrez and García-López (2012a) with sixth graders, efficiency of the attacking-the goal context differs considerably, as this is the context in which sixth graders achieved the lowest scores. Efficiency in the adaptation to the tactical contexts has been assessed in youth elite soccer players of different ages in González and collaborators’ studies (Under 9: González et al., 2012; Under 11: González et al., 2010; Under 13: González et al., 2011). Opposite to the present study, the younger soccer groups, U9 and U11, show better efficiency in 1A than in 2A, achieving the worst scores in attacking-the-goal (3A). The U13 soccer players scores were partially congruent with this study as they scored better in 2A than in 1A, but with efficiency of the attacking-the goal context (3A) being the context where U13 soccer players achieved the lowest scores, as it occurred in the study by Gutiérrez and García-López (2012a) with PE students of the same age (11–12 years old).

In the studies mentioned above, along with the findings in the present study, there seems to be a pattern associated to the age of the participants in regard to the tactical awareness related to 1A versus 2A. While soccer players and physical education students under 11 years old show better understanding of the tactical context that involves keep-the-ball (1A), soccer players and physical education students older than 11, score better in the tactical context that involves penetrate-the-defense. It seems that better understanding of the context that drives the objective of the game gives evidence of game understanding maturation, which may provide valuable information for physical education teachers and coaches on different understanding and awareness according to students and players. In this regard, the pedagogical principle of modification-exaggeration can be used for adjusting the task constrains to the different stages of learning.

Positive results in penetrating-the-defense contexts were also achieved in specific technical-tactical skills: passing and getting free. In penetrate-the-defense contexts, players showed very high decision-making in passing and getting free efficiency, as opposed to the scores in keeping-the-ball contexts. These results are congruent with findings based on a study conducted by Sanchez-Mora and colleagues (2011), where fourth graders scored significantly higher in passing and dribbling, both in decision-making and skill execution, when the action took place in the penetrating-the-defense context rather than in the keeping-the-ball context.

It is also noteworthy that participants achieved very high results in decision making in numerous defensive and offensive technical-tactical skills, while obtaining low execution scores in defensive technical-tactical skills execution. Comparing with studies involving low skilled players, as those by French and Thomas (1987) and Nevett et al. (2001), decision-making results were unexpectedly high. Although subjects in this study were older than those that participated in the aforementioned studies, it can be argued that this high decision-making performance and the differences with the reported studies could be also caused by the features of the game (e.g., game modifications and exaggerations). The design of the game in this study aimed to facilitate decision making through reducing the skill execution difficulties. On the other hand, the low execution scores on defensive technical-tactical skills execution, are consistent with those achieved by novices (Gutiérrez et al., 2011); they may be also caused by the lack of a systematic training process, and because during physical education lessons, the focus of interest is directed primarily at the offensive components of the game (Blomqvist et al., 2005). Discrepancy between efficiency in decision-making and efficiency and the success of the action can have negative effects in the learning process, because then, the feedback received by the subject informs only about the lack of success of the action, which impels the student to reinforce the behaviour, even though it was correct in terms of decision-making.

There was a high correlation between decision-making and execution in many technical-tactical skills. From this correlation, it can be inferred that in the majority of actions, success in execution was conditioned in a positive way by a correctly-made decision and, on the contrary, when the decision made was incorrect, the execution had more possibilities of not being successful. This is congruent with the findings of previous studies that demonstrated the importance of teaching and learning both skill execution and decision-making (French et al., 1996). These results are in line with the pedagogical principle of modification-representation, as its key point is to keep the information-movement coupling of the structured game (Tan et al., 2012), avoiding simplifications of the game that imply the lost of the essence of the actual game.

When grouping the game result analysis into global variables, it was observed that the decision making score was substantially better for the onthe-ball technical-tactical skills (*p*<.01) in comparison with off-the-ball scores. The same level of significance was presented in the execution in favour of the attack skills. These results are similar to those reported by Blomqvist et al. (2005). In that study, players reached the best results in decision making for on-the-ball offensive situations. These findings indicate that the egocentric play of the participants was not only related with the aim of playing the ball or get a goal as soon as possible, but also with the ability to perform better actions related to the ball and with the goal. Bayer (1992) placed the child behaviour in the first stages of sports learning, oriented to the goal and highly linked to the ball, because of an egocentric and goal-oriented way of play. It is possible that the maintenance of an egocentric way of play, a characteristic traditionally linked to childhood, in students as old as 14 years old, was due to the lack of experience. Global variables, which reflected the lowest scores, were execution in defensive actions (*M* = 34.69%; *SD*: 16.01) and execution in off-the-ball actions (*M* = 47.41%; *SD*: 19.72); execution onthe-ball (*M* = 49.44%; *SD*: 12.01); and decision making off-the-ball (*M* = 47.41%; *SD*: 19.4). To improve the game performance of participants, the teacher should design tasks following the pedagogical principle of exaggeration-modification. Those tasks should facilitate the success in those game aspects where lowest results were showed.

The results presented great importance of the cognitive aspects in game performance, and the necessity to take into account the different tactical contexts, both for the evaluation and teaching processes. To further extend the significance of cognitive understanding in game performance, future research should focus on the defensive side of the ball, particularly to explore the potential influence of the quality of the defense in decision-making efficiency.

Based on the results, a learning progression could be designed. To be congruent with pedagogical principles of GCAs (Mitchell et al., 2003; Oslin and Mitchell, 2006), the learning progression should be based on the following:

- Tactical problems are the determinant in tactical decision-making of performance, so they should be the principles that guide the progression.
- The decision making component and tactical problems are the elements games and sports learning progression should be based on.
- Movements and skills should be trained in game situations that address a specific tactical problem.
- Skill execution training should be subordinated to its use during the actual game.
- Based on the Mitchell et al.’s (2003) proposal, the progression could contemplate three levels. Although these authors establish one level for each age or grade (Mitchell et al., 1994), with a detailed assessment, as shown in this paper, each level could include three levels. Levels should be ordered beginning from those movements and skills that players have performed better in the decision making component in a specific tactical problem. This way the learning process would begin from those elements that the students are developmentally prepared to work in.

Table 3 presents the learning progression based on the results and the criteria described.

Blomqvist et al. (2005) reported that their study had two limitations, the first was the skill heterogeneity of the participants, and the second was that all of them were boys. We tried to avoid these two limitations by proper selection of the participants. First, we decided to research both girls and boys and second, we used a questionnaire to learn about their previous experiences, so that we would recruit less skillful students. However, high values in standard deviation were found in most of the variables, and therefore, subjects in our study had skill heterogeneity as well. In this regard more research is needed to explore the nature of these individual differences.

## Figures and Tables

**Table 1:** Description of the dependent variables to measure decision making

**Skill execution and Level 1 Decision-making: Technical-tactical skill selection**	**Level 2 Decision-making: Tactical-context-adaptation**
**Attacker on-the-ball**	Global-context-adaptation performance (Global efficiency during the whole game in adapting the actions to the tactical context)
Pass	
Shoot	
Moving with the ball	
**Attacker off-the-ball**	1A1A. Tactical-context-adaptation performance to keep the ball contexts (efficiency in selecting actions to keep the ball when the tactical context is coded as “keeping-the-ball context”)
Get free	
**Defender-on-the-ball**	2A2A. Tactical-context-adaptation performance to penetrating-the-defense contexts (efficiency in selecting actions to penetrate the defense when the tactical context is coded as “penetrating-the-defense”)
Mark (on-the-ball)	
Blocked shot	
Tackle	
Double team (on-the-ball)	
**Defender-off-the-ball**	3A3A. Tactical-context-adaptation performance to attacking-the-goal contexts (efficiency in selecting actions to try to score when the tactical context is coded as “attacking-the-goal context”)
Mark (off-the-ball)	
Interception	
Double team (off-the-ball)	
**Global variables**	Spectator-player (a player is coded as “spectator player” when he or she does not show tactical intention nor involvement on the game)
Defense/attack	
On-the-ball / off-the-ball	

**Table 2:** Percentage of effectiveness in the offence and defence variables

**Offensive variables**	**Actions (Average)**	**Decision making**	**Skill execution**
	**Attacker on-the-ball**		
Control			73.79(21.68)
Pass (total)	9.93(3.8)	89.24(20.17)	69.63(27.01)
Pass in 1A		64.81(44.44)	67.59(36.43)
Pass in 2A		93.38(15.39)	72.84(28.61)
Shoot (total)	1.84(2.29)	93.8(12.14)	80.31(25.65)
Shoot in 3A			84.96(22.89)
Moving with the ball / dribbling (total)	5.42(5.96)	88.49(16.61)	85.39(18.04)
Moving with the ball / dribbling in 1A		93.75(17.67)	87.48(19.41)
Moving with the ball / dribbling in 2A		88.47(18.53)	87.5(18.49)

	**Attacking player off-the-ball**		
Get free (total)	28(6.98)	72.52(18.48)	60.02(19.82)
Get free in 1A		65.63(25.74)	58.68(30.17)
Get free in 2A		73.58(19.65)	60.04(20.83)

	**Defender to attacker on-the-ball**		
Mark	10.37(9.41)	80.61(18.9)	24.52(27.09)
Blocked shot	2.42(2.71)	93.59(11.23)	10.4(14.62)
Tackle	1.47(1.47)	75.38(36.35)	29.49(37.98)
Double team	0.58(1.02)	92.86(18.89)	46.43(46.61)

	**Defender to attacker off-the-ball**		
Mark	26.37(7.51)	64.91(19.28)	44.22(22.22)
Interception	1.69(1.77)	86.92(29.82)	53.08(42.26)
Double team	3.32(2.81)	62.21(37.27)	30.72(34.4)

Note. SD in parentheses

**Table 3:** Proposal of learning progression based on the game performance assessment results

Player	Tactical problem	Level I	Level II	Level III
		*Effectiveness >*85%	*Effectiveness 70%–85%*	*Effectiveness <70%*
			Control	
Attacker on-the-ball	1A. Keeping the ball	Moving with the ball		Pass
	2A. Penetrating the defense	Pass Moving with the ball		
	3A. Attacking the goal	Shoot		
	
Attacker off-the-ball	1A. Keeping the ball			Get free
	2A. Penetrating the defense		Get free	
	
Defender-on-the-ball		Mark	Interception	
		Blocked shot		
		Double tam		
	
Defender-off-the-ball		Interception		Mark
				Double team
